# Percutaneous retrieval of right intracardiac mass with Inari Flow-Triever System

**DOI:** 10.1093/ehjcr/ytae315

**Published:** 2024-07-30

**Authors:** Maria Chiara Gatto, Achille Gaspardone, Fabrizio D'Errico, Gennaro Sardella, Massimo Mancone

**Affiliations:** Cardiology Unit, Emergency Department, Sant’Eugenio Hospital, ASL Roma 2, Piazzale dell'Umanesimo 10, 00144 Rome, Italy; Cardiology Unit, Emergency Department, Sant’Eugenio Hospital, ASL Roma 2, Piazzale dell'Umanesimo 10, 00144 Rome, Italy; Cardiology Unit, Emergency Department, Sant’Eugenio Hospital, ASL Roma 2, Piazzale dell'Umanesimo 10, 00144 Rome, Italy; Department of Clinical, Internal, Anesthesiology and Cardiovascular Sciences, ‘La Sapienza’ University of Rome, Rome, Italy; Department of Clinical, Internal, Anesthesiology and Cardiovascular Sciences, ‘La Sapienza’ University of Rome, Rome, Italy

The treatment of intracardiac masses is a complex clinical scenario. We describe two cases of right intracardiac masses with high embolic risk treated with a thrombo-aspiration system. The first case involves a 30-year-old male on dialysis for bilateral nephrectomy and previous percutaneous closure of an atrial septal defect, presenting with massive pleural empyema, methicillin-resistant *Staphylococcus aureus* bacteraemia, and a floating mass in the right atrium occupying the tricuspid orifice (*Panel A* and Supplementary material). The second case involves a 72-year-old woman with single chamber pacemaker and diabetes, admitted after a head injury following a dizziness episode. During hospitalization, bilateral deep vein thrombosis, bilateral pulmonary embolism, and a thrombus in the right atrium adjacent to the pacemaker lead were detected (Supplementary material). In both cases, the floating thrombus-like masses were considered at high embolic risk and the patients underwent to emergency percutaneous thrombo-aspiration treatment.^1–3^ After few attempts, the clots were successfully removed in cath-lab using the Inari Flow-Triever System (*Panel B* and Supplementary material). The first specimen had a cauliflower-like shape (*Panel C*) and resulted positive for methicillin-resistant *S. aureus*, therefore antibiotic therapy was optimized. The second specimen was a long red thrombus (*Panel D*), and the laboratory analysis was negative. The post-procedural course in both cases was free of complications, and both patients survived. The thrombo-aspiration systems have been designed for the removal of endovascular thrombotic formations and not specifically for the removal of endocavitary vegetations or masses. In particular, the Inari Flow-Triever System has been designed for rapid thrombus removal for patients with acute pulmonary embolism. However, our case demonstrates how it is technically feasible to aspirate atrial masses when the risk of embolization is very high. The successful management of these cases suggests the possibility that in emergency situations, the use of thrombo-aspiration systems can be useful in the removal of right intracardiac thrombotic masses.^4^

**Figure:**
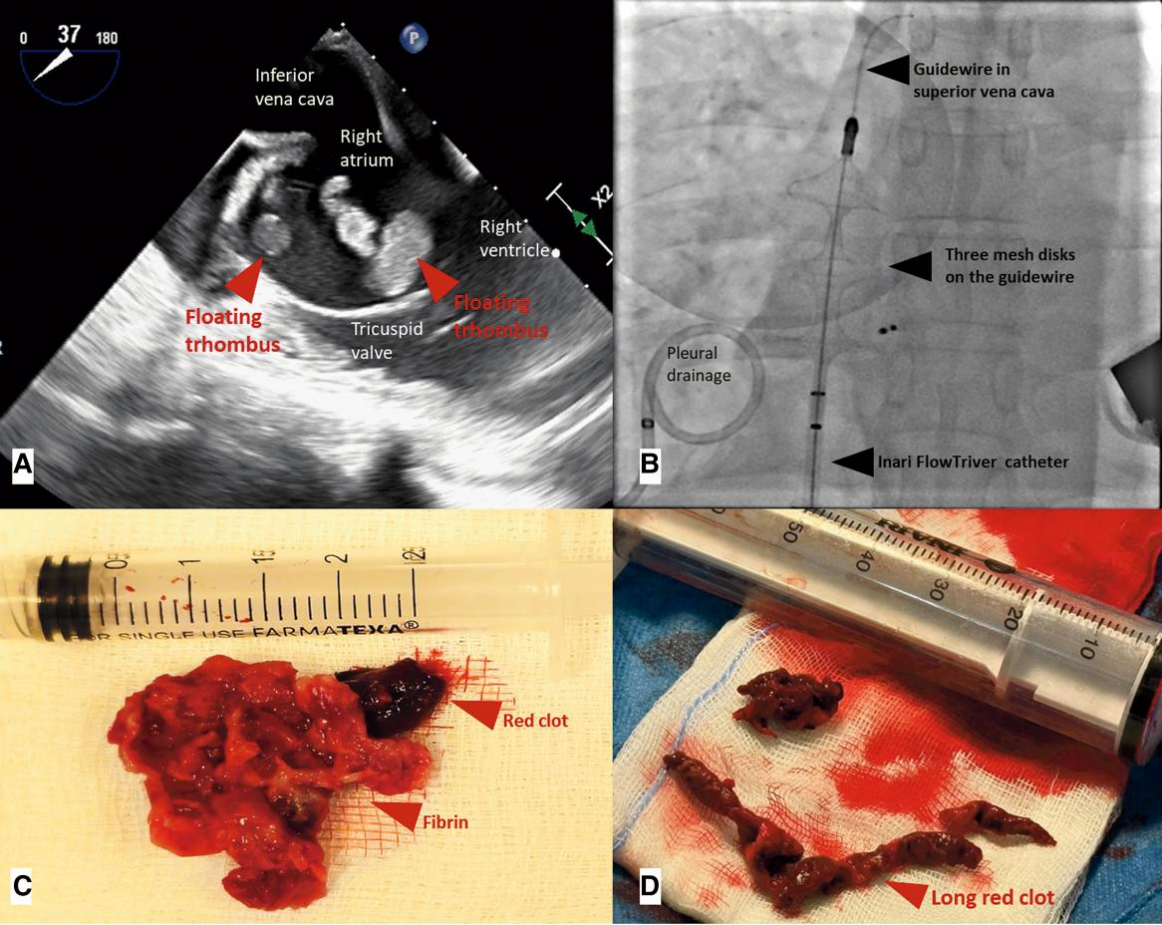


## Supplementary Material

ytae315_Supplementary_Data

## Data Availability

The data underlying this article are available in the article and in its online Supplementary material. A motivated request for additional data or information can be forwarded to the corresponding author.

## Supplementary material

**Consent**: The authors confirm that written consent for submission and publication of this case report, including images and associated text, has been obtained from the patients in line with COPE guidance.

**Funding**: None declared.
