# Supplementary material for: Percutaneous retrieval of right intracardiac mass with Inari Flow-Triever System
Source: Eur Heart J Case Rep. 2024 Jul 30;8(7):ytae315. doi: 10.1093/ehjcr/ytae315 (PMC11288366; doi:10.1093/ehjcr/ytae315)
Supplement: ytae315_Supplementary_Data [file ytae315_supplementary_data.zip › Suppl data rev.docx]

**SUPPLEMENTARY DATA**

**Clip 1:** First case transesophageal echocardiography showed a large multilobulated mass, with maximum diameter of 3.5 cm, originating from the superior vena cava and protruding into the right atrium up to the tricuspid plane (Clip 1 Suppl. data).

**Clip 2:** Second case transesophageal echocardiography showed a voluminous and serpiginous thrombotic formation, with a maximum diameter of 6 cm, adjacent to the pacemaker lead and protruding from the right side of the interatrial septum into the right ventricle during diastole.

**First Case retrieval procedure’s details**

Considering the elevated embolic risk, we decided to remove the thrombotic mass by using off-label the Inari FlowTriever System 24 French (Inari Medical, Irvine, CA, USA), an over the wire retrieval/aspiration system. The system consists of a support cable to which three nitilol alloy discs of different diameters are attached distally which are opened downstream of the thrombotic formation to block and withdraw it into the guiding catheter. In order to increase the suction force, a syringe in which a vacuum is made is attached to the guiding catheter. The system was inserted through a 33 cm long Gore DrySeal 24 French introducer positioned through the right femoral vein. The guiding catheter was initially placed across the tricuspid valve to obtain complete adherence to the thrombotic mass and to facilitate the aspiration of the floating thrombus through the valve. Several extraction attempts were carried out using discs of different sizes (from 15-18 mm to 19-25 mm). Each attempt was associated with blood aspiration using the syringe connected to the guiding catheter. Only a few tissue fragments were extracted from this attempt. Assuming that the mass was adherent to the lateral atrial wall in continuity with the inflow tract, the guiding catheter was then repositioned in the superior vena cava, and the thromboaspiration maneuver was performed again (Figure 1 Suppl. data). After repeated attempts, the floating mass was eventually extracted.


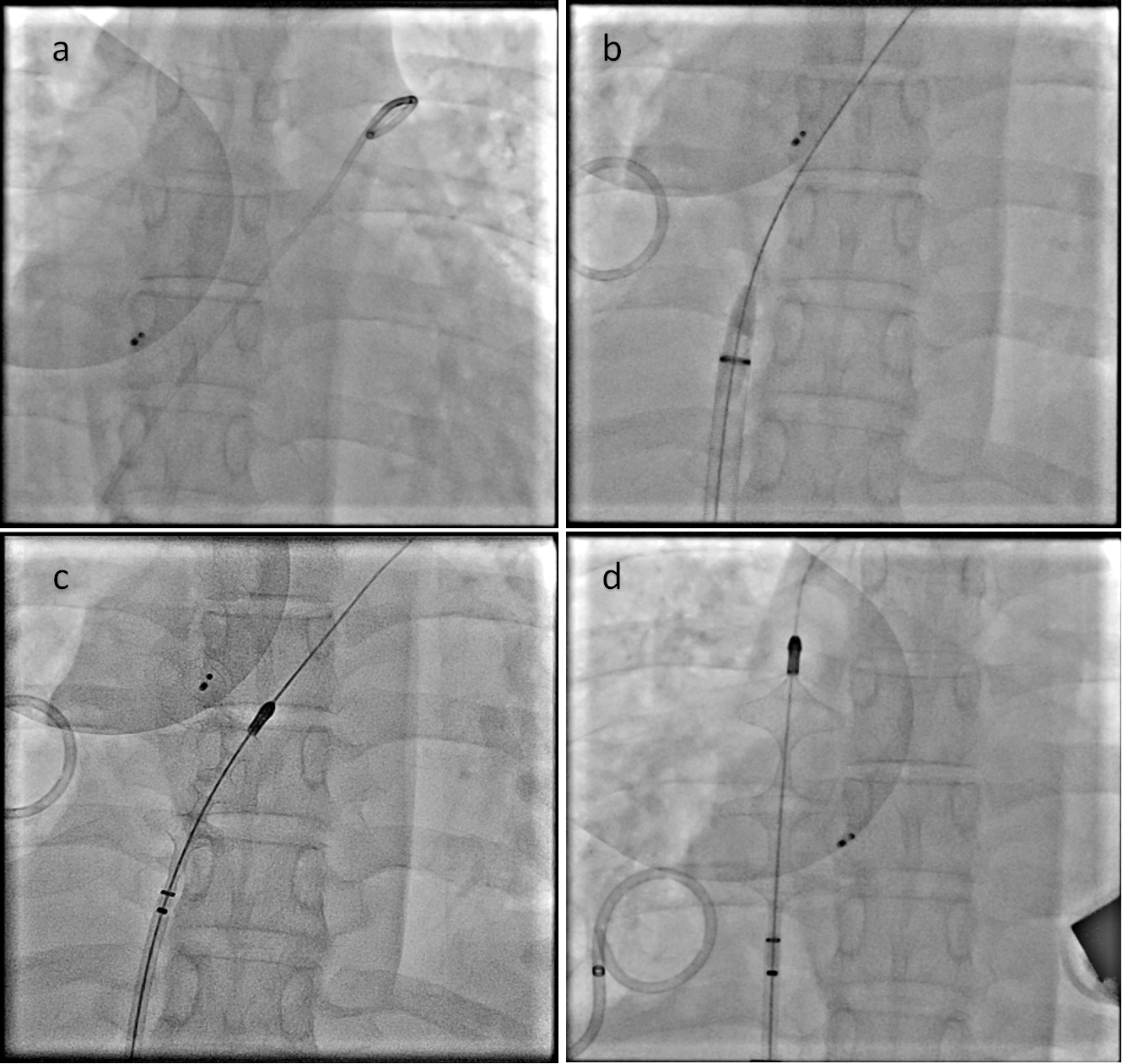


#### Figure 1 Suppl. data: Intracardiac thrombus retrieval procedure. A: Placement of a pigtail catheter in the pulmonary artery; B: Exchange with a stiff guide and insertion of the suction system at the level of the tricuspid valve; C: Opening of the three mesh disks adjacent to the tricuspid valve; D: Opening of the three mesh disks on the guidewire positioned in the superior vena cava. The asterisk indicates the drainage catheter placed in the pleural space.

**Second case retrieval procedure’s details**

Due to the prohibitive operative risk, the heart team excluded the open surgery approach and we scheduled the patient for a percutaneous removal of the thrombotic formation using the FlowTriever system. A guidewire was inserted through the left jugular vein into the right atrium. After that, we replaced it with a stiff guidewire that was advanced into the pulmonary artery and the right ventricle using a Pig Tail. However, despite multiple attempts at thromboaspiration with 20 French FlowTriever, we were unsuccessful. Therefore, we obtained left femoral venous access and advanced another stiff guidewire into the pulmonary artery. We then performed multiple thromboaspiration using 24 French FlowTriever. The last attempt successfully removed the thrombotic fragments opening the three mesh disks adjacent to the pacemaker lead. An inferior vena cava filter was placed as a final step. Afterwards, follow-up echocardiography showed that most of the thrombotic material adhered to the pacemaker lead had disappeared (Figure 2 Suppl. data).


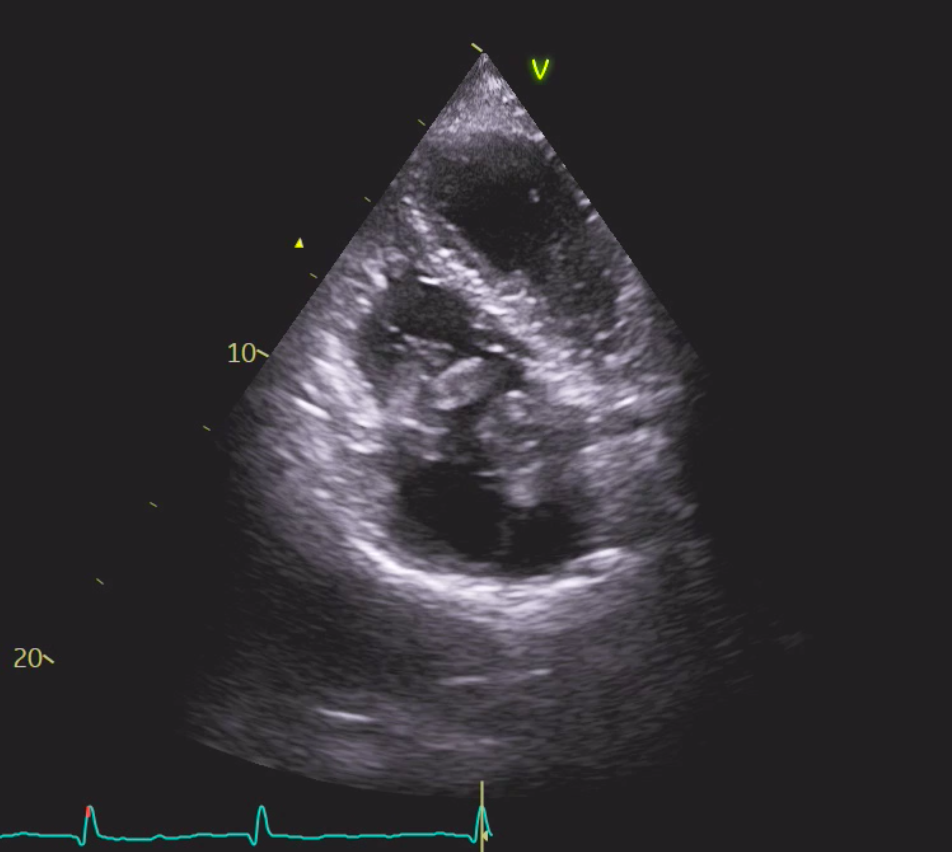

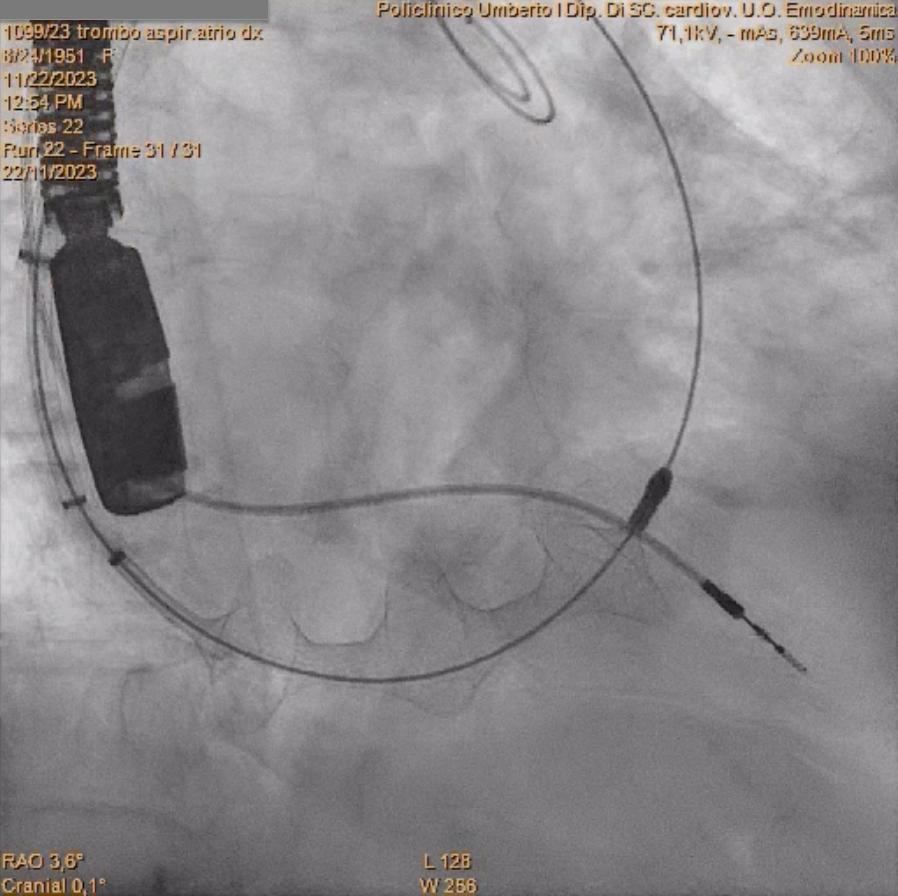

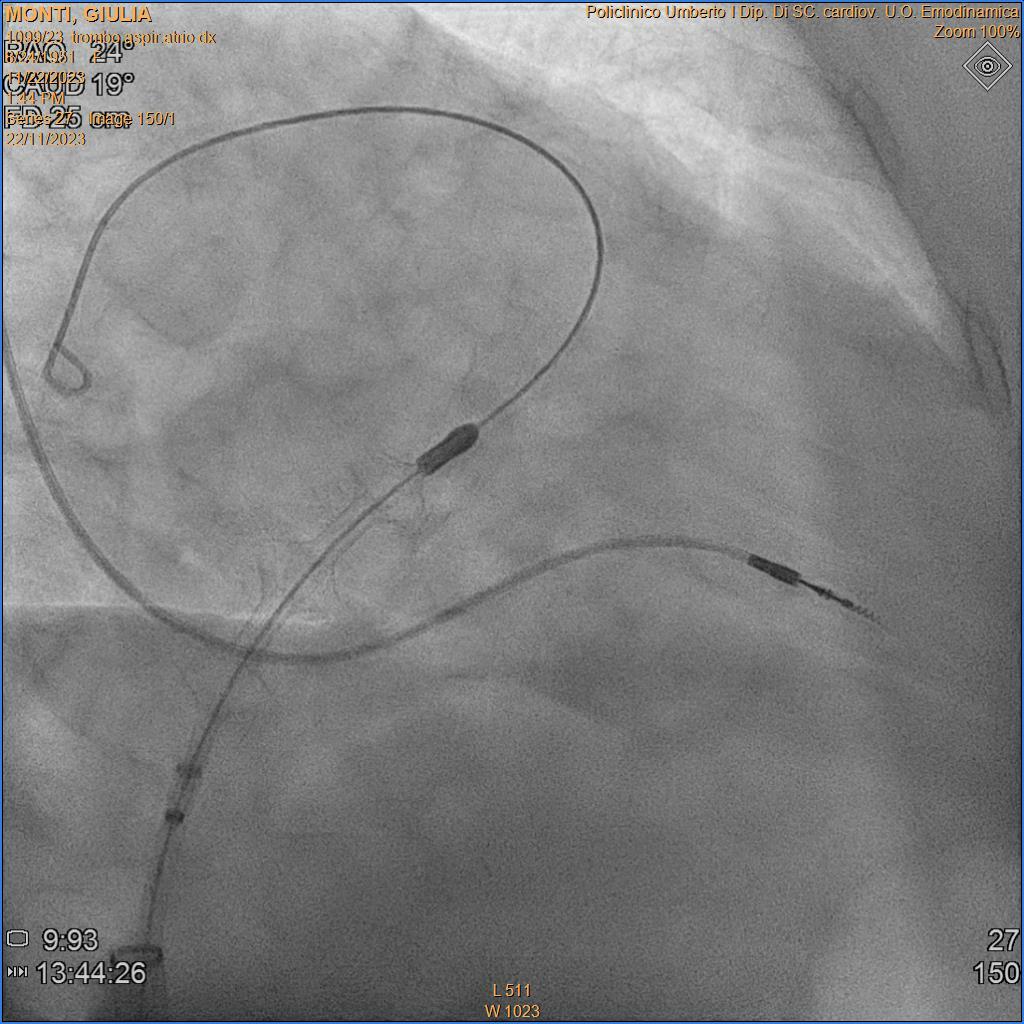

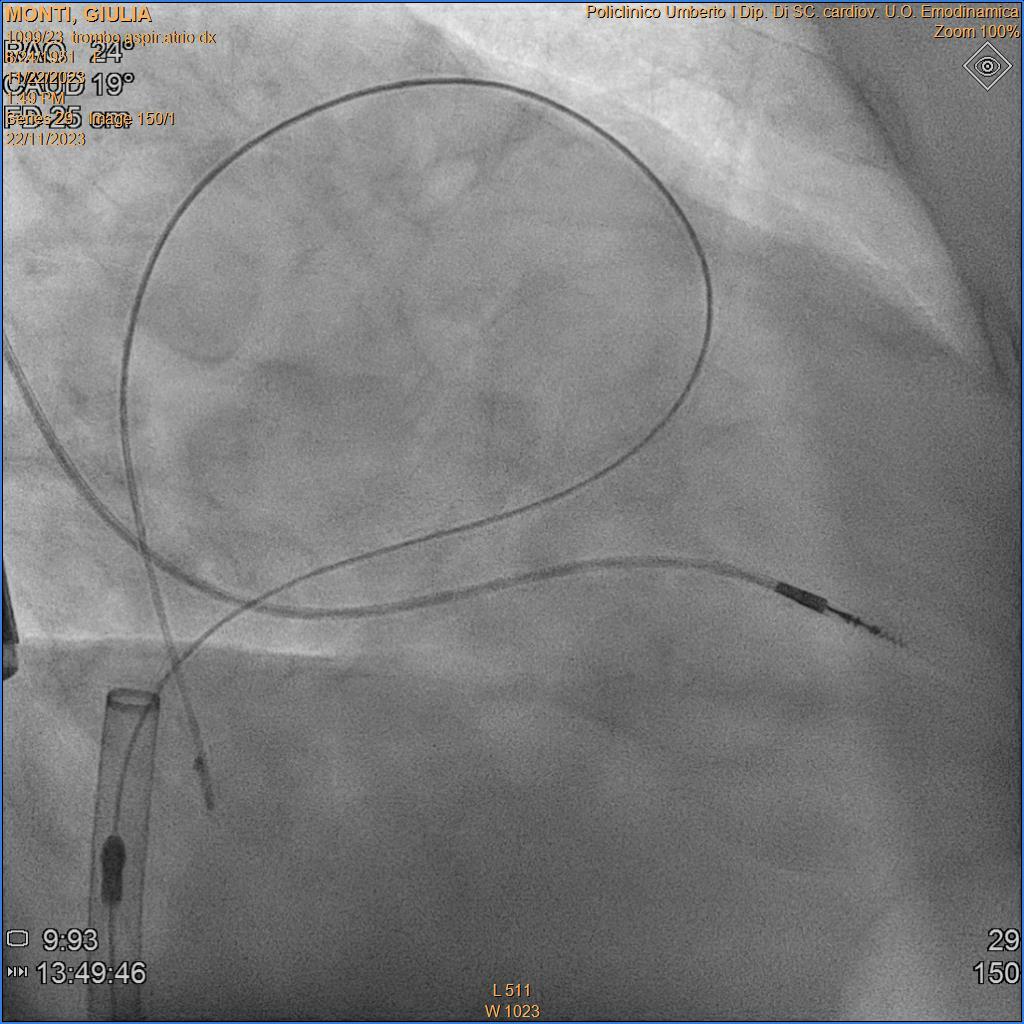

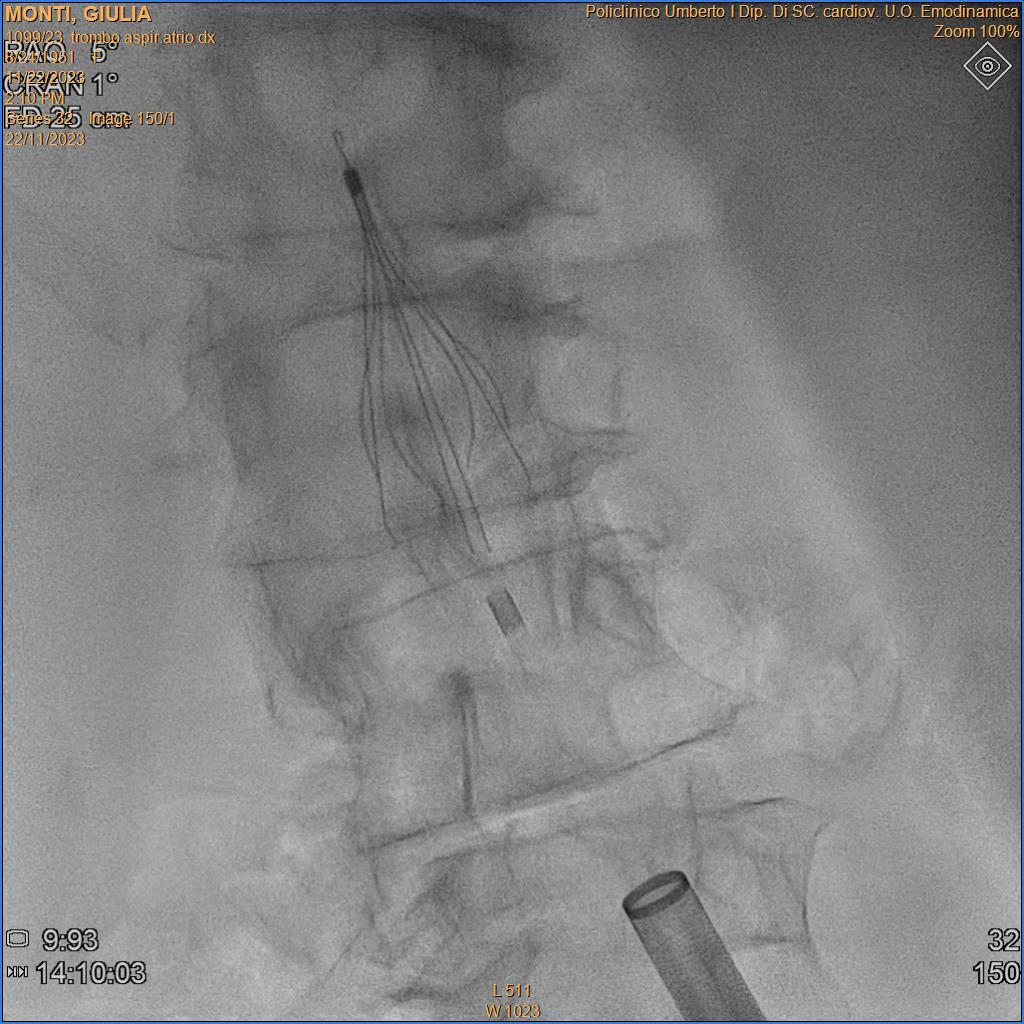

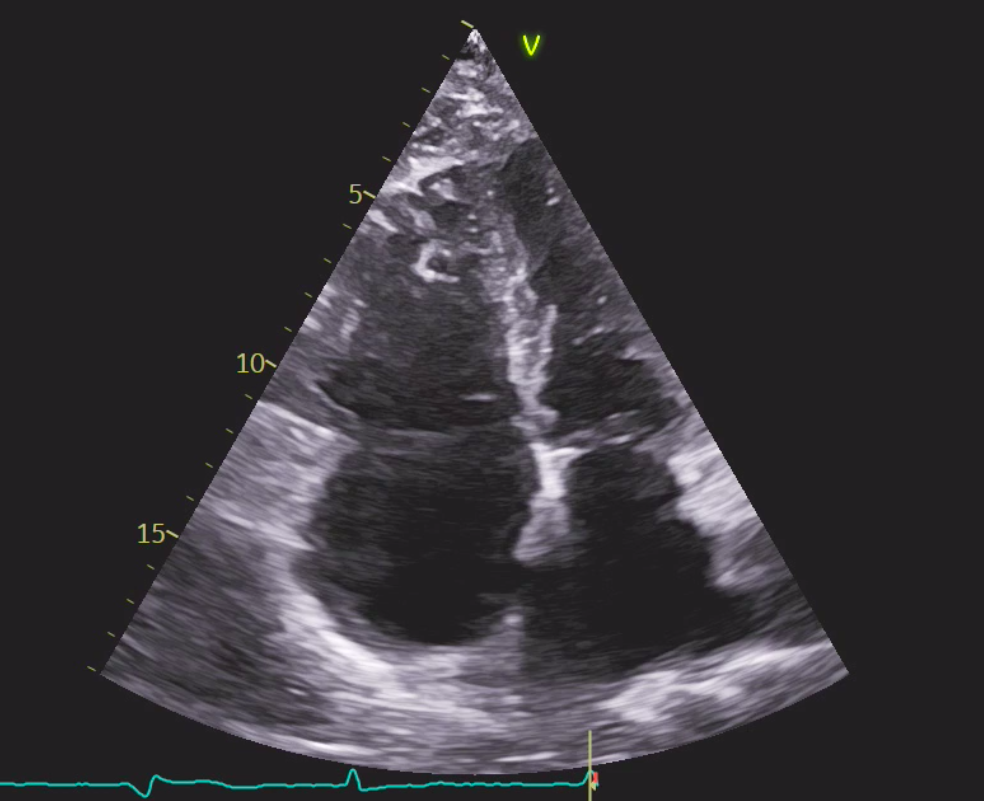


b

a

c

d

e

f

#### Figure 2 Suppl. Data: A: Floating thrombus in the right atrium. B: Unsuccessful attempt at thromboaspiration via the left jugular vein using 20 Fr FlowTriever System. C: Insertion of the suction system via the left femoral vein after placing a pigtail catheter in the pulmonary artery and exchanging it with a stiff guide at the level of the tricuspid valve; D: Successful attempt at thromboaspiration via left femoral vein using 24 Fr FlowTriever System and opening the three mesh disks adjacent to the pacemaker lead ; E: Inferior Vena Cava Filter placement; F: Follow-up echocardiography.
